# The INTERPRET Decision-Support System version 3.0 for evaluation of Magnetic Resonance Spectroscopy data from human brain tumours and other abnormal brain masses

**DOI:** 10.1186/1471-2105-11-581

**Published:** 2010-11-29

**Authors:** Alexander Pérez-Ruiz, Margarida Julià-Sapé, Guillem Mercadal, Iván Olier, Carles Majós, Carles Arús

**Affiliations:** 1 Centro de Investigación Biomédica en Red en Bioingeniería, Biomateriales y Nanomedicina (CIBER-BBN). Spain; 2 Departament de Bioquímica i Biologia Molecular. Universitat Autònoma de Barcelona, UAB. Cerdanyola del Vallès, 08193, Spain; 3 Institut de Biotecnologia i de Biomedicina (IBB). Universitat Autònoma de Barcelona, Barcelona, Spain; 4 Institute of Neuroscience. Universitat Autònoma de Barcelona, Barcelona, Spain; 5 Department of Radiology. Institut de Diagnòstic per la Imatge (IDI) Centre Bellvitge. Hospital Universitari de Bellvitge. L'Hospitalet de Llobregat, 08907, Spain; 6 School of Psychological Science, The University of Manchester. Manchester, UK

## Abstract

**Background:**

Proton Magnetic Resonance (MR) Spectroscopy (MRS) is a widely available technique for those clinical centres equipped with MR scanners. Unlike the rest of MR-based techniques, MRS yields not images but spectra of metabolites in the tissues. In pathological situations, the MRS profile changes and this has been particularly described for brain tumours. However, radiologists are frequently not familiar to the interpretation of MRS data and for this reason, the usefulness of decision-support systems (DSS) in MRS data analysis has been explored.

**Results:**

This work presents the INTERPRET DSS version 3.0, analysing the improvements made from its first release in 2002. Version 3.0 is aimed to be a program that 1^st^, can be easily used with any new case from any MR scanner manufacturer and 2^nd^, improves the initial analysis capabilities of the first version. The main improvements are an embedded database, user accounts, more diagnostic discrimination capabilities and the possibility to analyse data acquired under additional data acquisition conditions. Other improvements include a customisable graphical user interface (GUI). Most diagnostic problems included have been addressed through a pattern-recognition based approach, in which classifiers based on linear discriminant analysis (LDA) were trained and tested.

**Conclusions:**

The INTERPRET DSS 3.0 allows radiologists, medical physicists, biochemists or, generally speaking, any person with a minimum knowledge of what an MR spectrum is, to enter their own SV raw data, acquired at 1.5 T, and to analyse them. The system is expected to help in the categorisation of MR Spectra from abnormal brain masses.

## Background

The diagnosis of an abnormal brain mass usually depends on the histopathological analysis of a brain biopsy, since imaging techniques can only correctly characterize the type and grade of a tumour in a few instances [1]. Proton magnetic resonance spectroscopy (^1^H-MRS) is one of several MR-based techniques, which gives information about metabolites in solution in the millimolar range of concentration, in living tissues. The MR spectroscopic pattern has been shown to be characteristic of certain tumour types, but since there is no specific marker signal for type or grade and several signals change at a time in pathological conditions. Therefore, the need for a multivariate analysis of the MR spectrum for diagnostic or prognostic purposes has been pursued for nearly two decades [2,3]. The INTERPRET project [4] successfully developed a program for brain tumour characterisation with the use of ^1^H-MRS data: it was named "INTERPRET decision-support system (DSS)". It involved the accrual of a large number of MRS data from brain tumour patients, the creation of a database [5], the training of a mathematical classifier [6] and finally, the introduction of both data and classifiers into the final program. The performance of the MR spectrometers of INTERPRET participants was assessed bimonthly by a short protocol that used a dedicated phantom [7]. After the end of the project, the DSS started to be distributed free of charge through the project's web page http://gabrmn.uab.es/INTERPRET. However, it presented several practical limitations with respect to its routine use at radiology facilities. First of all, since database development, population and DSS development had been performed in parallel, the final system did not benefit from the extensive data quality controls applied to the database [5], and therefore the DSS contained small errors. The most important limitations still, were the lack of connection between the data processing that is always needed with MRS data and the introduction of new cases of unknown pathology for their evaluation. Therefore, the system in its first version could only be used for demonstration purposes or as an MRS learning tool, but not as one that allowed users to enter a new, unprocessed MR spectrum from any format or manufacturer and to evaluate it with the system.

This paper explains the path followed from 2003 to 2010, from the first release of the system to the current 3.0, to turn the DSS into a program that 1^st^, can be easily used with any new case from any MR scanner manufacturer and 2^nd^, improves the initial analysis capabilities of the first version.

## Implementation

### Data types stored

The system can store the following data types for each case:

-Processed Single Voxel (SV) MR Spectra acquired at 1.5 T, at short (20-32 ms) and long (135-144 ms) TE, including information on TE, TR and acquisition sequence (PRESS or STEAM).

-MRI (T1-W (weighted), T2-W, proton density (PDW) and volume of interest (VOI) images). Accepted formats are jpg, bmp and DICOM http://medical.nema.org/, which are displayed with ImageJ http://rsbweb.nih.gov/ij working in the background.

-Clinical information (Clinical Record, CR) [4]: Age, Sex, Tumour Location, Tumour Size, Radiological Diagnosis, Total Tumour Removal, Subtotal Tumour Removal, Stereotactic Biopsy, Paraffin Section WHO Classification [8], Daumas-Duport Astrocytoma Grade [9], Outcome Score at Three Months, Outcome Score at Two Years, Concomitant Disease, Histopathology Validated, Localisation Validated and Assigned Class.

-Case notes.

All cases included were acquired with INTERPRET-compatible acquisition protocols [4].

The term "tissue type" is used instead of the terms "tumour class" or "disease", since the system was designed to handle not only distinctions among different tumour types but also between diseases or regions of the brain (such as normal non-affected brain or diseased brain).

### Database

**Figure 1:**
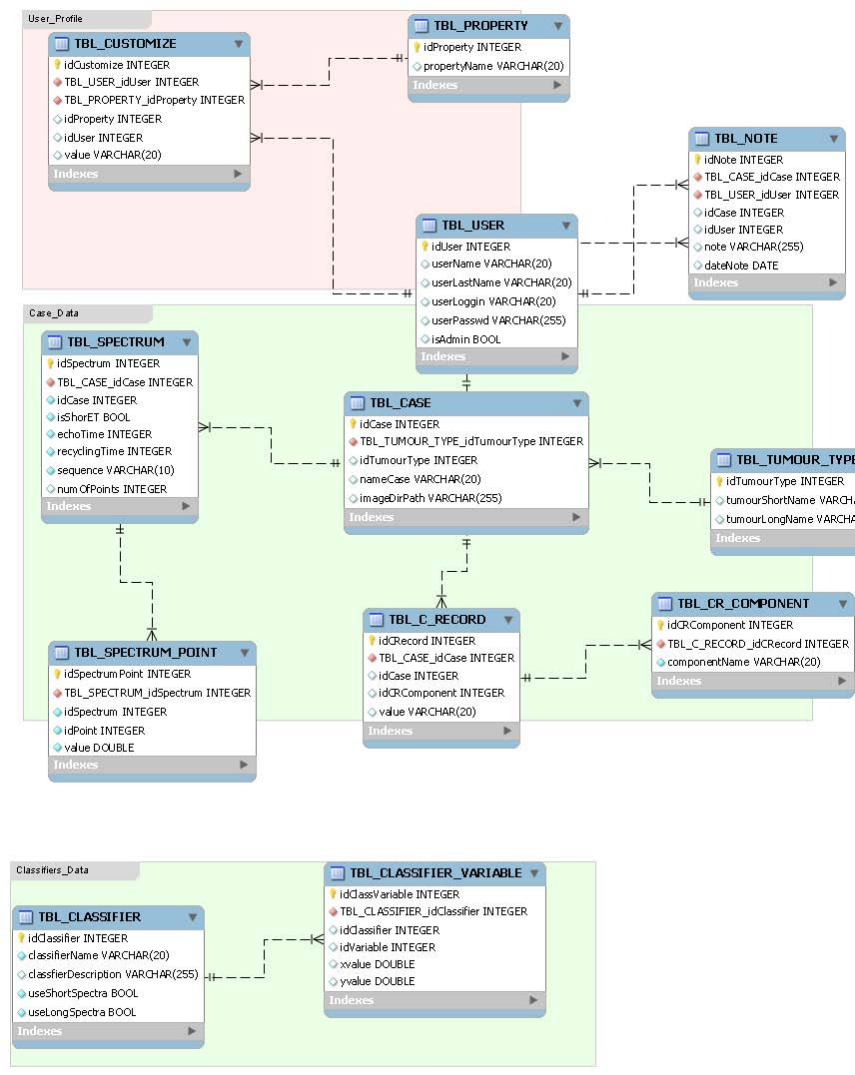
**E-R diagram of the embedded database**. The database stores Users' profiles, Cases' data and Classifiers' data. The "User" table handles usernames and passwords, as well as administration rights. The "Case" table depends from the "User" table and stores the disease type, case ID and where the information objects are stored. There are three dependent tables, Spectrum, Clinical Record and Tumour type, which store information related to acquisition parameters and MR spectra file formats, clinical information and the list of possible diseases, respectively. Finally, the Classifier table handles which classifier and echo times the system has to choose for display. The Classifier variable table stores × and Y positions of each case in the 2D plot, according to the classifiers selected.

Version 3.0 comes with an embedded database developed in SQL over the standalone HSQLDB database engine http://www.hsqldb.org. The database stores not only the cases and their related information but also classifier information, users' profiles and their customisations. Figure 1 shows the entity-relationship (E-R) diagram of the database.

### The data manipulation software

The Data Manipulation Software (DMS) was incorporated into the INTERPRET DSS on version 2.0. It is an MR spectra-processing tool developed during INTERPRET (2000-2002) with a set of routines from mMRUI http://sermn02.uab.cat/mrui which, 1^st^) automates the MR spectra processing procedure and 2^nd^) generates what is called the "INTERPRET canonical format" or DMS format, which allows multi-scanner, multi-format spectral analysis [4]. This constitutes a relevant feature since different scanner manufacturers provide different number of points and sweep widths (range of frequencies covered), yielding variable spectral resolutions (points/ppm).

Since 2002, the range of formats available in the radiological environment increased, especially for General Electric (GE), which is now on 15.x and 20.x versions depending on the countries. The DMS is only able of reading up to 6.x GE Probe raw data files and 8.x and 9.x Probe raw data files provided that they carry a SAGE Header companion file (SHF) and the .7 suffix is eliminated. To our knowledge, in 2010 the DMS can still perform the whole pipeline (from format reading to delivery of the automatically processed and aligned spectrum) for Philips raw data files.

**Figure 2:**
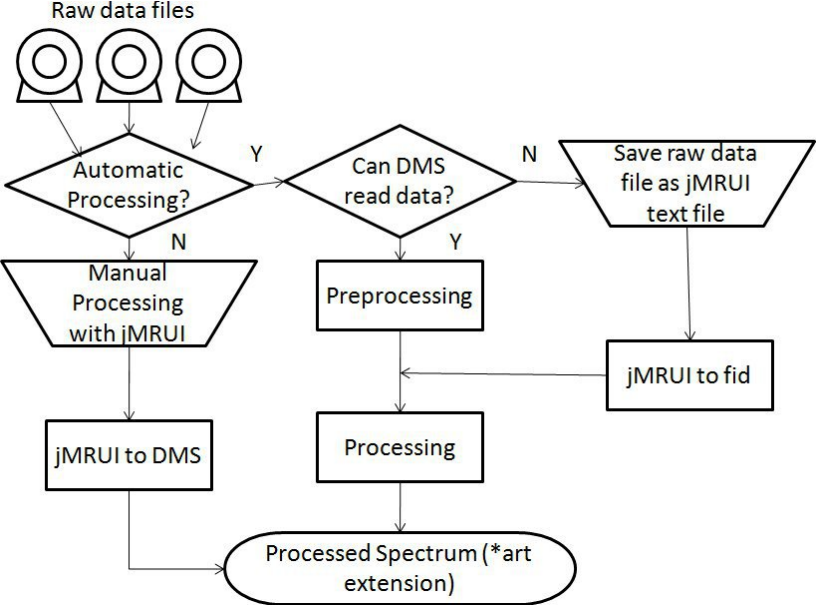
**Obtaining processed MRS data in the DMS format**. There are two ways to obtain the processed MRS data, manually processing them with jMRUI or with the DMS. The operation is divided in two steps: preprocessing and processing. Preprocessing identifies the format and converts files to a canonical raw format that is subsequently processed by the DMS. The DMS can read some formats and in that case processing is automatic. If the format is not readable, then jMRUI should be used, either for performing the preprocessing or the processing. There are the jMRUI to canonical raw format (jMRUI2fid) and the jMRUI to DMS converter (jmrui2DMS).

Therefore, it was necessary to find a workaround for the problem of reading new vendor formats, and the Java based DMS (JDMS) was developed to this end. With the JDMS, the JMRUI program [10,11] can be used as a format converter. The user can open his/her raw data file and save it as text file without carrying out any processing. This is especially important for those raw data files from multi-channel coils where the files have to be manually consolidated in order to obtain a single acquisition file. In that case, each file (one for metabolites, one for water signals) has to be saved after adding all corresponding acquisitions, in text format with *.txt extension using the JMRUI. Finally, in acquisitions for which the automatic processing does not provide a satisfactorily aligned or phased spectrum, the user is encouraged to use JMRUI and the JDMS as well. In these cases, spectra should be processed with JMRUI using the same processing parameters used by the DMS, with minor zero or first order phasing and saved as JMRUI txt files. When these files are entered into the system, the embedded JDMS will automatically convert them into the DSS format. The processing parameters are fully described in the Help section of the software. Figure 2 summarises the different paths for processing raw data files to obtain a file in the DMS format.

The DMS format has 512 points to represent the range between 7.1 to -2.7 ppm http://gabrmn.uab.es/dms. It has the "art" extension and is a simple ASCII text file with real numbers (simple precision) separated by spaces, where the first point corresponds to the spectral intensity at 7.1 ppm.

**Figure 3:**
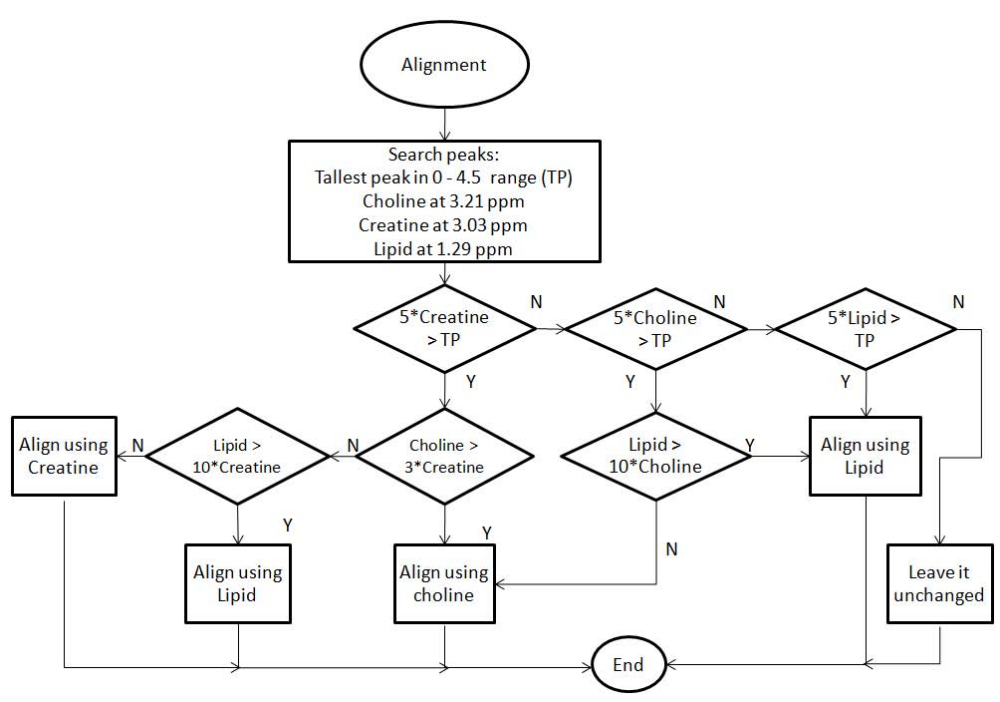
**Alignment algorithm**. The system searches for the tallest peak (TP) in the 0-4.5 ppm range and for the three important peaks, in this order: Creatine, Choline and Lipid. If the SNR of the interesting peak is lower than five times the SNR of the TP, then the following peak is analysed in order to be chosen as reference. At the end, if one of the peaks is the chosen one for alignment, the spectrum is aligned and the process is terminated, otherwise the spectrum is left unchanged.

The automatic processing performed by the DMS is essentially similar to what was already described in [4], with the following improvements:

1. Water filtering using HLSVD [12] with 10 lorentzians instead of 5.

2. Line broadening of 1 Hz instead of 0.8 Hz.

3. Baseline offset corrected taking into account both sides of the water peak (-2 to -1 ppm and 9 to 11 ppm).

4. Linear interpolation, for those file formats with an unequal number of points in the [7.1, -2.7] ppm interval with respect to Philips files [4].

5. Alignment: The alignment process adds zeros to the beginning or to the end of the spectrum and removes the same number of data points in the opposite side, in order to move the desired peak to the expected position. Alignment is based on the algorithm shown in Figure 3 and it searches, in this order, for the Creatine peak, the Choline-containing compounds peak, and the Lipid peaks, at 3.03, 3.21, and 1.29 ppm respectively. Each peak is searched in a range near the expected position (± 4 or 5 data points). A point will be considered a peak only if its intensity value is greater than the value of the contiguous points on its left and right sides. If none of these three peaks is found in the ranges expected, the spectrum cannot be automatically aligned and is left unchanged.

The DMS is distributed as well as an independent software module http://gabrmn.uab.es/dms.

### Case sets

The availability of an embedded database in version 3.0 allowed the inclusion of more than one case set.

Four different case sets are therefore available: 1) The INTERPRET cases, 2) The IDI-Bellvitge cases, 3) Example cases and, 4) User cases. If available, for each case set, it is possible to have the short TE spectrum, the long TE spectrum and the concatenated short+long TE spectra as in [13]. In addition, the four sets of cases can be displayed with any of the classifiers, one or all four at a time. A short description of each set follows below:

1) Cases from the INTERPRET database [5], checked for quality control and reprocessed with the DMS or JMRUI plus JDMS if necessary (for a detailed list of which cases had been processed with the DMS or with JMRUI+JDMS, see the program's help). The case numbers and the superclasses they belong to are shown on Table 1. Note that for the INTERPRET set, two types of cases are available, those used to train the classifiers (Most common tumour types, 217 cases at short TE and 195 cases at long TE and at short+long TE) and those from the database which belong to other, less common classes (87 cases at short TE and 71 cases at long TE or short and long TE).

**Table 1:** DSS 3.0 Datasets (number of cases) and classifiers they served to train.

				*Superclasses used for training the classifiers*					
***Dataset***	***Spectra available***	***Classification***		***Meningioma***	***Aggressive***	***Low-grade glial***	***Subtotal***	***Cases from other classes***	***Total***
								
		***problem***	***Method(s)***						

INTERPRET	Short TE (20-32 ms)	MCTT	LDA	58	124	35	217	87	304
	
	Long TE (135-144 ms)	MCTT	LDA	55	109	31	195	71	266
	
	Short + Long TE	MCTT	LDA	55	109	31	195	71	266

				***Pseudotumoural***	***Tumoural***	***Normal*** ***brain***	***Subtotal***	***Cases from other classes***	***Total***

IDI-Bellvitge	Short TE (30 ms)	T vs.PS	LDA	19	46	5	70	0	70
	
	Long TE (135 ms)	T vs.PS	LDA	19	46	5	70	0	70
	
	Short + Long TE	T vs.PS	LDA and Ratios[14]	19	46	5	70	0	70

Specifications of the two main datasets included in the system, the INTERPRET and the IDI-Bellvitge dataset. Each dataset has short and long TE spectra and both short and long TE spectra concatenated. Different classification problems have been analysed with these datasets. Furthermore, in the IDI-Bellvitge dataset, the same classification problem has been solved in two different ways, either by an LDA classification or by a peak height ratio-based classifier [14]. The INTERPRET dataset contains cases used for training the classifiers as well as from other less common types of tumours. Note that for INTERPRET the number of cases available at short and long TE is different. MCTT: Most common tumour types; T vs.PS: Tumour vs. pseudotumoural disease. See [4,13] for further details on superclass definition.

2) Cases from the Institut de Diagnòstic per la Imatge of the Bellvitge Hospital (IDI-Bellvitge) from Barcelona, Spain used in the [14] study, plus 5 normal volunteer cases from the same centre, obtained in the context of INTERPRET [4]. The distribution of cases is shown on Table 1. All these cases come with short and long TE MR spectra and without any other clinical or MRI information, except for the definitive diagnosis that is indeed included. Inclusion criteria for that study were 1) presence of an untreated, solid, nonnecrotic brain mass suggesting a brain tumour, 2) diagnosis of pseudotumour or glial tumor grades II or III of the WHO confidently established, 3) spectra available obtained at both short and long TE, and 4) the spectra of good quality at visual inspection. The diagnosis of pseudotumour was based on clinical and imaging follow-up. From a clinical stand-point, patients had an acute to subacute onset of signs or symptoms involving a focal neurologic deficit mimicking the findings of an intracranial neoplasm. Imaging follow-up ranged between 2 and 77 months and showed reduction or resolution of the mass. The diagnosis of brain tumour was considered to be confidently established when a sample of the tumor could be evaluated and the pathologist could establish a single diagnosis. Histology slides were not circulated to consulting pathologists like in INTERPRET.

3) Two example cases are available for user practice. These come from the INTERPRET project as well but had not been used for classifier development.

4) New cases can be added through the "Load New Case" option in the "User Cases" menu on top of the screen. If the user stores them into the database, they become "User Cases". All users in the centre using the same DSS installation can see them but the uploader is the only user allowed to edit them. This set is initially void after the DSS installation.

### Quality control of data for classification

All information related to INTERPRET cases was checked for consistency (See "Access to the INTERPRET-validated database" link in http://gabrmn.uab.es/INTERPRET).

MRS data: This is an especially sensitive issue, since the MR spectra processing protocol might affect classification. Previously to classification, each case was processed with the DMS wherever possible and after that, it was individually checked by expert spectroscopists for alignment and phasing. For each case in the INTERPRET set, classification was performed using the MRS data and their histopathological diagnoses, which were counterchecked against the INTERPRET database [5]. After inclusion of each dataset in the GUI, a quality assurance protocol as in [5] was performed to ensure traceability of the INTERPRET dataset in the DSS to the INTERPRET database. When available, MRI and clinical information were checked as well. All cases were also checked for quality control parameters (signal-to-noise (SNR) > 10 and water linewidth < 8 Hz as computed in [4]) reprocessed with the DMS or JMRUI plus JDMS if necessary (old cases dating from the 1990's period, in which there was no unsuppressed water file available, and those in which it was necessary to perform a minor phasing adjustment).

MRI and clinical data: A quality assurance protocol as in [5], was followed for the release of version 1.1, checking against the INTERPRET database for MRI and clinical data correctness.

### Available classifiers

In its current version 3.0, two abnormal brain mass classification problems are addressed. The system handles data at short TE (20-32 ms), long (135-144 ms) and concatenated short and long TE spectra [13]. One of the most commonly changed parameters in clinical MR spectroscopy is echo time (TE), since it can give different information about the metabolites contributing to the spectral pattern and hence, affect the classification problem addressed [15-17].

The two abnormal brain mass classification problems addressed are summarised below and on Table 1:

A) *Distinction among most common tumour types (MCTT)*, as in the original INTERPRET DSS [4]. The classifiers were trained with the following brain tumour superclasses: low-grade meningiomas, low-grade glial tumours (astrocytomas, oligodendrogliomas and oligoastrocytomas of WHO grade II) and high-grade aggressive tumours (glioblastomas and metastases). Three different classifiers are available, i.e. short, long and concatenated short+long TE. The classifiers are based on linear discriminant analysis (LDA).

B) *Distinction between tumour and pseudotumoural disease (T vs. PS)*.

B1) Classifies among pseudotumoural disease, tumours and normal brain tissue. The individual diagnoses available are, for pseudotumoural disease, Acute Infarct, Multiple Sclerosis, Acute Disseminated Encephalomyelitis, and "Non Specific Pseudotumoural Disease"; for tumoural disease, Astrocytoma WHO grade II, Oligodendroglioma WHO grade II, Oligoastrocytoma WHO grade II, Astrocytoma WHO grade III, Oligoastrocytoma WHO grade III. Normal brain tissue cases were taken from the INTERPRET project [5] (healthy volunteers). The INTERPRET protocol requested that for normal volunteer spectra the volume of interest was selected just above the ventricles in such a way that it contained mostly white matter avoiding grey matter and cerebral spinal fluid [7], obtaining a mean spectral pattern for all volunteers corresponding to the expected for normal white matter [7].

Three different LDA-based classifiers are available, i.e. short, long and concatenated short+long TE.. Data used were single-centre retrospective, and are described in [14].

B2) Another classifier option addresses the same problem with the same cases set, but with a ratio-based decision rule based on peak heights. A graph based in the Choline/N-acetyl Aspartate ratio at short TE vs. the m-Inositol/N-acetyl Aspartate at long TE is used according to [14].

The six LDA-based classifiers were developed with the "SpectraClassifier" software (http://gabrmn.uab.es/sc, [18]). Relevant features were selected in the 4.05 - 0.01 ppm range using the Sequential Forward method. Features were validated using the correlation-based criterion. Classification was performed with Fisher Linear Discriminant Analysis and evaluation was performed with the bootstrap method. The optimum number of features was selected following criteria described in [19] and the use of an independent test set.

For the MCTT classifiers, the test set consisted in an independent set from three medical centres: Centre Diagnòstic Pedralbes (CDP), Institut d'Alta Tecnologia (IAT) and Institut de Diagnòstic per la Imatge (IDI)-Badalona in Barcelona, Spain (data not available with the software), processed in the same conditions as above. The test set used was composed of 63 short and long TE spectra: 3 low-grade meningiomas, 20 low-grade glial tumours and 40 high-grade aggressive tumours. For the T vs. PS classifiers, the test set was the same as in [14]. Table 2 shows the classification results for all the classifiers included in the different releases.

**Table 2:** Classification results of the LDA-based classifiers included in the different versions of the INTERPRET DSS.

Version	Classifier	TE	Accuracy with training set	Classifier tested with a test set	Number of cases in the test set	Accuracy with test set
1.1	MCTT	Short	94.00%	yes	87	85.05%

2.0	MCTT	Short	88.94%	no	-	-

2.0	MCTT	Long	82.56%	no	-	-

3.0	MCTT	Short	89.00%	yes	63	82.54%

3.0	MCTT	Long	84.20%	yes	63	69.84%

3.0	MCTT	Short+Long	89.20%	yes	63	82.54%

3.0	T vs.PS	Short	85.50%	yes	19	78.95%

3.0	T vs.PS	Long	81.10%	yes	19	84.21%

3.0	T vs.PS	Short+Long	92.10%	yes	19	78.95%

MCTT: Most common tumour types; T vs.PS: Tumour vs. pseudotumoural disease. Summary of classifiers released.

### Overview of the graphical user interface (GUI)

**Figure 4:**
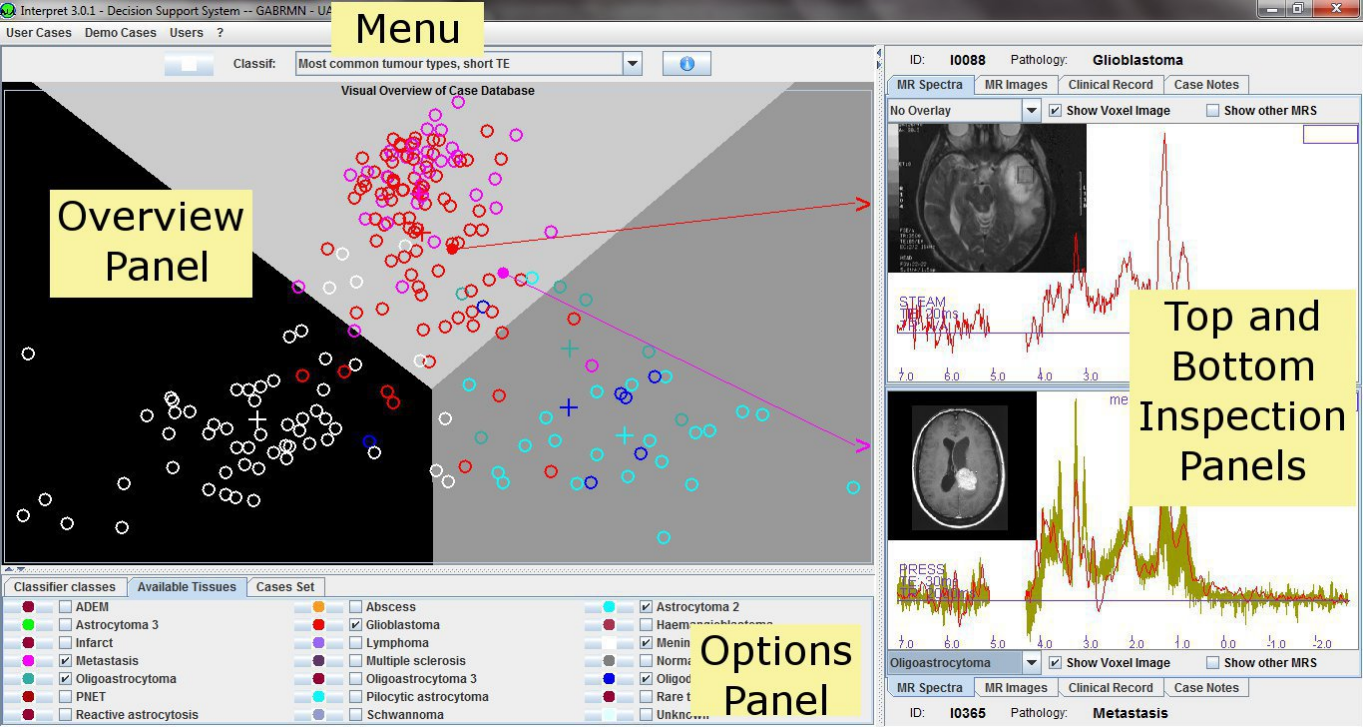
**INTERPRET DSS GUI**. The GUI maintains the same four parts since its first release. The Menu is located on top of the window and allows entering and managing both cases and users. There are four panels, which are now fully resizable. The Overview Panel, which is on the left side of the GUI, allows exploring the cases (coloured glyphs) that are distributed in a 2-D plot. The Options Panel is located below the Overview Panel and serves to customise the classifier classes, the available tissues or tumour classes and the cases sets, as well as the glyphs colours and sizes. The Top and Bottom Inspection Panels, on the right side, allow the user to examine two MR spectra and to visually compare them, and also to visually compare with mean and standard deviation plots for any of the classes of the chosen classifier

The INTERPRET DSS 3.0 preserves the layout of the first release. Figure 4 shows the system GUI, which is divided into two parts: The left side is the database explorer "Overview Panel" and the right side is the case explorer. The "Overview Panel" shows cases included into the embedded database, grouped by case sets and by tissue type or superclass. The right side is used to display individual cases, and two "Data Inspection Panels" are provided (top and bottom).

The first version only contained one case set (the INTERPRET dataset with spectra at short TE) and one classifier (for discriminating the most common tumour types at short TE [4]). Now, users can select one out of several classifiers through the top menu provided. When a classifier is selected, the left side adopts a predefined configuration that shows only the cases used to develop the classifier selected. Users can also select which case sets and classes will be shown in the "Overview Panel", with the help of the options provided in the "Option Panel" placed below it. The "Overview Panel" is a 2D scatter-plot of cases where each case is represented by a glyph. Cases are spatially positioned according to the selected classifier. The shape and colour of each glyph is fully customizable, and they represent the grouping criteria (classes, superclasses or tissue types). The cross indicates the position of the class, superclass or average pattern of the tissue type in a specific classifier.

The background colour of the "Overview Panel" is always the same, regardless of the classifier chosen. However, this colour can apparently change when the user chooses the "Show Boundaries" option from the popup menu. Choosing this option forces the panel to show the regions defined by the classifier boundaries. Each region is drawn with a predefined colour but can also be customised with the small buttons placed at the left side of the superclass name or the tissue type name in the respective tab of the "Options Panel".

The top menu "Classif:" allows not only to choose a classifier, but also to make a personalised overview, with the use of the "Make your own overview" option. Its layout has changed slightly in the last version and now there is a combo box. When selecting the "Make your own Overview" option, the user has to choose first if he/she wants to make a personalised overview based on short or on long TE spectra. After that, the ppm features can be selected (with the sliders provided) for creating a 2D display of peak heights or peak height ratios. Note that in order to avoid the "division by zero" error, when the user selects the ratios option, and with the purpose of improving the display of all cases in a set, the minimum value of all spectra in the chosen dataset is used as offset in order to turn positive all points in the spectra. The offset is dynamically recalculated in case of need (e.g. a new case). In this respect, the user should be aware that this might produce unexpected ratio plots when dealing with datasets at long TE with strong inverted lactate or alanine peaks.

Users can review cases in detail through the two "Data Inspection Panels" provided. MR spectra, MRI, CR and notes associated to the selected case can be examined using the respective tabs.

### Intended use

The user is expected to enter a new case formed by a 1.5 T SV MRS set (preferably one short and one long TE spectrum) from a patient with an abnormal brain mass, obtained under INTERPRET-compatible acquisition conditions [4]. After entering the new case into the system, the DSS is to be used as an analysis tool for the SV MRS set. The analysis is expected to be performed using all the system capabilities, 1^st^) Selection of a classifier depending on the clinical question that the user may have, 2^nd^) Position of the SV MR spectrum in the "Overview Panel" of the classifier/s selected, 3^rd^) Analysis of the spectral pattern with the help of the two "Data Inspection Panels" provided, through comparison of the problem spectrum with neighbouring cases from the "Overview Panel" and 4^th^) Decision whether to permanently store the case or not. The ability to store a case is a new feature of the DSS and the user is able to study every new case through different executions of the program. The uploader will be able to edit any stored case at any time.

### Inserting additional classifiers and datasets into the system

**Figure 5:**
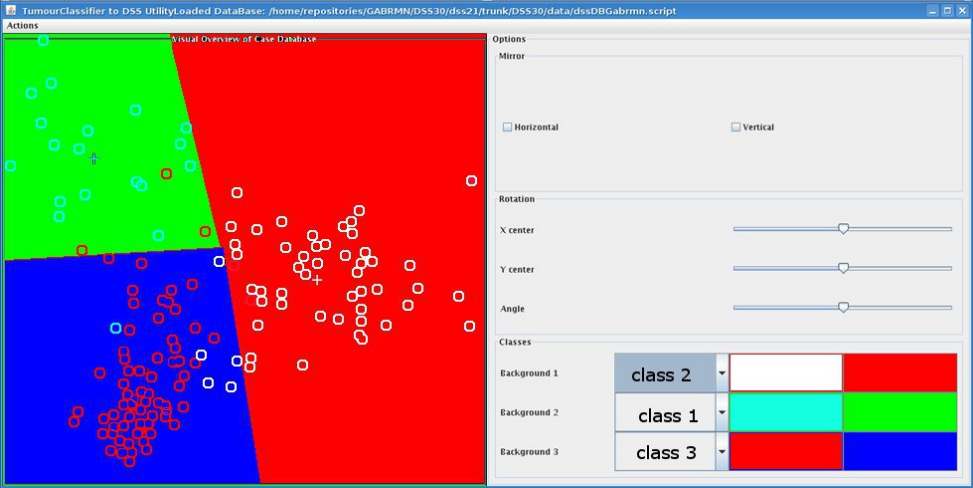
**The SC2DSS tool**. The module is divided in two panels, right and left. The right panel corresponds to the desired look-and-feel of the Overview Panel in the DSS 3.0 GUI. The left panel allows the developer to perform the visual tuning actions to fit the 2-D latent space of each classifier into the Overview Panel in the GUI. Three features can be tuned, the latent space can be horizontally or vertically flipped with the "Mirror" function (top right panel). It can also be rotated ("Angle" slider) and the centre can be translated either horizontally ("X center" slider) or vertically ("Y center" slider) to adjust the cases to the 2D space. The background colour of each class and their respective glyphs can be customised with the "Classes" panel at the bottom right of the screen.

Before a new classifier can be used in the DSS, it should be tailored to maintain the DSS look and feel. Characteristics like aspect ratio (width/height ratio), colours, names and so on can be customised with the tools included in the software called SC2DSS (Figure 5). SC2DSS is only available to the developers' team dss@gabrmn.uab.es and it allows to tailor and upload a new classifier into the INTERPRET DSS. Once a new classifier is developed the users will receive a new version of the database without having to download the whole DSS system again. This application can populate the embedded database with the dataset used to develop the classifier, taking into account the superclasses defined to create each classifier, the groups of tissue types and their names. Moreover, SC2DSS inserts the mathematical representations of the classifier and the classification boundaries represented by lines into the DSS-embedded database. In all previous DSS versions, boundaries between regions defined by classes were not those defined by classifiers, but the bisectors between the centroids of each class on display. For LDA classifiers on Version 3.0, the true boundaries defined by the LDA classifiers are displayed.

## Results

### Versions released

DSS development followed several steps in the 2003-2009 period until version 3.0, with emphasis in two different aspects: the quality control of the dataset and the improvements in the GUI.

**Version 1**. Released in December 2002. It is the initial development described in [4].

**Version 1.1**. Released in June 2004. The changes with respect to previous versions were:

1. Update of all information (clinical and spectroscopic) contained in the prototype, according to [5]. In this version, the DMS was still not embedded but the processing pipeline was refined to its current state. Baseline correction and automated alignment were implemented. A final quality assurance step was also performed to ensure correct migration of clinical and spectral data from the database to the prototype.

2. Retrained classifier at short TE, according to [4], with the updated information.

3. The classifier was tested with an independent set of 87 cases (12 low-grade gliomas, 58 aggressive tumours-glioblastomas and metastases- and 17 low-grade meningiomas) [18].

**Version 1.2**. Released in November 2004 [20]. In this, the DCM images were incorporated and an additional quality assessment on all images was performed.

**Version 2.0**. Released in July 2007. This version included for the first time a classifier for the long TE spectra of the INTERPRET set, in addition to the short TE spectra classifier. It also used the term "case" to designate the association of the short and long TE MR Spectra obtained from the same VOI during the same MR study. The "Manual Overview" was adapted for its use with long TE spectra. The selected case was kept selected even if the user changed between different views (classifier overview or manual overview) or classifiers (short or long TE). In this version, the DMS was finally incorporated into the DSS and the user could work with his/her own cases directly from the raw data files as it has been described previosly. A further quality control of the processed MR spectra was performed and some of them were reprocessed with the JMRUI+JDMS pipeline. Classifiers at short and long TE were retrained with an in-house MatLab^® ^pipeline, essentially equal to what will be described for version 3.0. The only difference was that the number of variables of the classifier was set to 13 both for the short and the long TE classifiers and that no independent test set was used to validate classifiers' performance.

**Version 3.0**. Released in September 2009. Changes with respect to previous versions: The system changed the storage strategy and contains an embedded database. The user can store his/her cases permanently. Different users can share "Case Notes" turning the system into a knowledge base. The look and feel of the GUI has been made fully customisable with respect to colours and glyphs. The following new concepts have been incorporated: possibility of having different data sets, case label by superclasses, classifier boundaries, user profiles, multiple classifiers and concatenated short and long TE spectra on display and for building the classifiers. The embedded database allows semiautomatic incorporation of new datasets and classifiers without requiring any further change into the GUI. Two more releases, 3.0.1. (November 2009) and 3.0.2 (January 2010) account for minor Windows Vista and 7 compatibility issues and the DMS distribution, respectively.

## Discussion

In this work we have shown how the initial INTERPRET DSS was improved in several aspects while maintaining the same look and feel. The improvements have been gradually released in successive versions in the 2003-2010 period, and can be categorised in three different aspects: GUI enhancements; increased analysis capabilities, and data quality and assessment checks.

Although several published approaches to the automated characterisation of MR spectra from abnormal brain masses do exist [21-24], especially for multi-voxel spectra, the INTERPRET DSS is, to our knowledge, the only system that helps users analyse and classify their own SV MR spectral data obtained at a field strength of 1.5 T. As in the initial systems' conception, the software does not provide an answer, but is aimed to help users extract information from data, basically through comparison with similar cases. The comparison is directed by the classifiers available. In contrast to the first version, where only one classifier had been implemented, the current one incorporates seven classifiers, accounting as well for several acquisition conditions. In this regard, potential users of the system can also check previous literature on advice for choosing the most suitable classifiers [25-27].

There is currently no consensus on the best TE for classifying among brain tumours, some previous work has shown [13,17] that short TE could be better for its improved sensitivity to mobile lipids and heavily J-modulated or short T2 compounds. In any case, the use of the information from both TEs should also be evaluated, and the system now provides the tool for this. The introduction of classifiers for distinguishing whether a brain mass is a tumour or not can be of interest in some situations where the MRI is inconclusive [14]. Although the classifiers available have been evaluated with independent test sets, the system as a whole may benefit from an extensive clinical evaluation that ideally would have to consider the following aspects:

1^st^) Usability. Whether the different degrees of medical or spectroscopic expertise influence how the system is used and the conclusions that are extracted. Now, users can choose among 7 classifiers and can also make their own overviews. The system does not recommend any classifier over another. Therefore, which one is to be chosen? Should this vary with the clinical problem addressed? Is the radiologist the one suggesting the classifiers to be used? Is the medical physicist or the radiology technologist the one to decide how to make the analysis?

2^nd^) Transferrability. Whether the classifiers provided have similar performances when challenged with cases from different clinical centres.

3^rd^) Classification performance limits. Whether the classifiers provided fulfil the range of clinical questions in abnormal brain mass characterisation. Furthermore, there are several discriminations with clinical importance that have not been accounted for in any of the DSS versions: Glioblastomas vs. Metastases, or Lymphomas vs. Other WHO grade IV tumours. The Glioblastoma vs. Metastasis discrimination problem has been previously attempted [4,15,28,29], but results are not easily generalisable and multivoxel spectroscopy has been previously shown to be better than SV at this distinction [30]. The system is now prepared to incorporate new classifiers and databases, therefore from version 3.0 onwards the introduction of a new classifier and its associated database should not be a problem as long as it could be viewable through a 2D or even a 3D plot. There is one additional limitation of the system that will also need to be addressed in the future: Since 2006, users of previous DSS versions have been declaring interest in the possibility to enter and analyse MRS data acquired with 3 T scanners, which are becoming more and more frequent. The SC2DSS tool will allow to overcome this limitation. However, it remains to be tested whether the current INTERPRET canonical format of 512 points adequately represents the information contained in the raw data from different manufacturers and equally importantly, whether classifiers generated at 1.5 T perform well with 3 T data, and under which circumstances e.g. for which classification questions.

The successive versions of this system have been distributed to some 150 centres throughout the world. It is expected that the use of version 3.0 and feedback from users will help to shape and further improve future versions of the INTERPRET DSS.

## Conclusions

The INTERPRET DSS 3.0 allows radiologists, medical physicists, biochemists or generally speaking, any person with a minimum knowledge of what an MR spectrum is, to enter their own SV raw data, acquired at 1.5 T, and to analyse them. The system is expected to help in the categorisation of MRS from any abnormal brain mass.

## Availability and requirements

Project name: INTERPRET Decision Support System version 3.

Project home page: http://gabrmn.uab.es/dss.

Operating system(s): Windows XP, Vista 7.

Programming language: Fortran, C, C++, Java.

Other requirements: Java 1.5 or higher.

License: With disclaimer signature.

Any restrictions to use by non-academics: All users need the license agreement.

## Abbreviations

^1^H-MRS: Proton magnetic resonance spectroscopy; CR: Clinical Record; DCM: Dicom, Digital Imaging and Communications in Medicine; DMS: Data Manipulation Software; DSS: Decision support system; E-R: Entity - Relationship; GUI: Graphical User Interface; HSQLDB: HyperSQL Database; INTERPRET: International network for Pattern Recognition of Tumours Using Magnetic Resonance; JDMS: java-based DMS; jMRUI: java-based Magnetic Resonance User Interface; LDA: Linear Discriminant Analysis; MCCTT: Most common tumour types; mMRUI: Matlab-based Magnetic Resonance User Interface; PD: proton density; PRESS: point-resolved spectroscopy; SC2DSS: SpectraClassifier to DSS; SQL: Structured Query Language; STEAM: stimulated-echo acquisition mode; SV: Single voxel; SNR: Signal-to-noise ratio; T vs. PS: Tumour vs. Pseudotumour; T1W: T1-weighted MR images; T2W: T2-weighted MR images; TE: Time of echo or echo time; TR: Recycling time; VOI: Volume of interest; WHO: World Health Organisation

## Authors' contributions

Study design and coordination: CA, MJS, APR. Manuscript drafting: APR, MJS, CA. Software development and integration: v1.1 and 1.2, GM, v2.0 onwards: APR. Classifier training: v1.1 and 1.2, GM, v2.0, IO, v3.0, MJS. JDMS: GM, IO. All authors read and approved the final manuscript.
